# Preoperative vertebral fracture assessment reveals bone fragility missed by DXA in patients undergoing total hip arthroplasty

**DOI:** 10.1186/s42836-026-00419-1

**Published:** 2026-08-12

**Authors:** Yudai Yano, Yoshinobu Uchihara, Sachiko Kawasaki, Munehito Seko, Masakazu Okamoto, Tadashi Fujii, Kenji Kawamura

**Affiliations:** 1 Department of Orthopaedic Surgery, Nara Medical University, Nara, 634-8521 Japan; 2 Department of Orthopaedic Surgery, Kashiba Kouseikai Hospital, Nara, 639-0222 Japan

**Keywords:** Total hip arthroplasty, Dual-energy X-ray absorptiometry (DXA), Bone mineral density, Vertebral fracture assessment, Osteoporosis, Preoperative assessment, Bone fragility

## Abstract

**Background:**

Bone fragility assessment prior to total hip arthroplasty (THA) is typically based on bone mineral density (BMD) measured by dual-energy X-ray absorptiometry (DXA). Meanwhile, prevalent vertebral fracture (VF), an important indicator of bone fragility, may be present even when BMD does not meet the diagnostic criteria for osteoporosis. Although preoperative bone health assessment has become crucial in patients undergoing THA, VFs remain insufficiently evaluated. This study aimed to investigate the prevalence and distribution of DXA-defined osteoporosis and VFs in patients undergoing THA and to evaluate how often DXA alone fails to detect clinically relevant bone fragility.

**Methods:**

This retrospective observational study included all consecutive patients aged ≥ 45 years who underwent primary THA between January 2021 and December 2025. Those undergoing THA for non-osteoarthritis etiologies were excluded. BMD was measured at the lumbar spine and femoral neck using DXA, and prevalent VFs were assessed on preoperative lateral thoracolumbar radiographs using Genant’s semi-quantitative method. DXA-defined osteoporosis was defined as a T-score ≤ − 2.5 at any measured site. Clinical osteoporosis was defined as DXA-defined osteoporosis and/or VF. The prevalence and distribution of DXA-defined osteoporosis, VF, and their overlap were assessed. Multivariable logistic regression analysis adjusted for age, sex, body mass index, and contralateral hip status was performed to identify factors associated with VF without DXA-defined osteoporosis.

**Results:**

Among the 352 included patients, DXA-defined osteoporosis was identified in 84 (23.9%) and VFs in 59 (16.8%). Clinical osteoporosis was observed in 118 patients (33.5%). Of these, 34 patients (9.7% of the total cohort; 28.8% of those with clinical osteoporosis) had VFs without DXA-defined osteoporosis and would not have been identified using DXA alone. Advanced age was independently associated with VF without DXA-defined osteoporosis (adjusted odds ratio, 2.65 per 10-year increase; 95% confidence interval, 1.65–4.24). In patients aged 80 years or older, the prevalence of VF without DXA-defined osteoporosis was 30.6%, and that of clinical osteoporosis was 58.3%.

**Conclusions:**

DXA alone underestimated clinically relevant bone fragility in patients undergoing THA. Preoperative radiographic evaluation of VF may improve bone fragility assessment, particularly in older patients.

## Background

Hip osteoarthritis (OA) is one of the most prevalent musculoskeletal disorders, affecting approximately 36 million people worldwide according to the Global Burden of Disease Study; this number is projected to reach 60 million by 2040 due to population growth and aging [1]. Accordingly, the number of total hip arthroplasties (THAs) performed worldwide has steadily increased [2]. Furthermore, indications for THA have expanded to include elderly patients because of improvements in implants and surgical techniques [3]. The average age of patients undergoing THA has steadily increased in recent years [3–5]. Although THA is a highly successful procedure for relieving pain and restoring hip function [6], it carries several risks of complications related to bone fragility, such as periprosthetic femoral fracture (PPF), which is a major reason for revision surgery after THA [7]. With the increasing age of patients undergoing THA, bone fragility-related complications have become crucial issues in this population. Therefore, an appropriate assessment of bone fragility in patients with hip OA is essential to ensure surgical safety and postoperative outcomes.

Conventionally, osteoporosis is diagnosed based on bone mineral density (BMD) measured in the hip or lumbar spine using dual-energy X-ray absorptiometry (DXA) [8]. Although low BMD is widely recognized as a major risk factor for fragility fractures, a history of fragility fracture is also adopted as an important factor in fracture risk assessment tools [9]. Moreover, according to the diagnostic criteria of the Japanese Society for Bone and Mineral Research (JSBMR), fragility fractures are included in the diagnostic criteria for osteoporosis [10]. Vertebral fractures (VFs) have long been recognized as the most common osteoporotic fractures, accounting for up to half of all fragility fractures [11]. According to the JSBMR guidelines, the presence of VF itself constitutes a diagnosis of osteoporosis, regardless of BMD values [10]. Population-based studies have demonstrated that VFs are highly prevalent [12–14]. For example, the ROAD study reported a prevalence of approximately 19% among women in a community-based cohort with a mean age of 65 years [12]. In contrast to hip fractures, many VFs are asymptomatic and can only be detected on imaging; approximately one-quarter to one-third of VFs are clinically symptomatic [15–17]. In addition, VFs frequently occur even in individuals whose BMD does not meet the diagnostic threshold for osteoporosis [18, 19]. Therefore, the radiographic evaluation of VFs plays an important role in the assessment of bone fragility, in addition to BMD measurements.

Currently, bone fragility assessments in THA patients are largely based on BMD. The prevalence of osteoporosis among patients undergoing THA is approximately 20–24% based on BMD measurements [20, 21]. However, the prevalence of clinically relevant bone fragility when VFs, important indicators of bone fragility, are considered remains unclear in this population. Therefore, this study aimed to investigate the prevalence of DXA-defined osteoporosis and morphometric VFs in patients undergoing THA and to evaluate the distribution of bone fragility identified by these assessments. The identification of bone fragility overlooked by BMD assessment alone may improve perioperative risk assessment in this population.

## Methods

This retrospective observational study was approved by the institutional Ethics Committee (approval no. 2027-03-1-020). Between January 2021 and December 2025, consecutive patients aged ≥ 45 years who underwent primary THA performed by a single surgeon at a single institution were included. If a patient underwent staged bilateral THA during the study period, only the first THA was included. Patients who underwent THA following femoral neck fracture or for non-OA etiologies, including idiopathic osteonecrosis (ION), rheumatoid arthritis (RA), rapidly destructive coxopathy (RDC), and subchondral insufficiency fracture (SIF), were excluded.

All included patients underwent DXA (SONIALVISION G4, Shimadzu Corporation, Japan) of the lumbar spine (L2–L4) and bilateral femoral necks (FN) to measure BMD at the time of surgical decision-making. In patients with a history of spinal fixation, regions affected by instrumentation were excluded from the lumbar spine BMD assessment. If the contralateral hip had already undergone arthroplasty, FN BMD was obtained only on the operative side. Lateral thoracolumbar spine radiographs (T10–L5) were also obtained for all patients as part of the routine preoperative evaluation around the time of BMD assessment. Contralateral hip OA was defined on preoperative radiographs as Kellgren–Lawrence (KL) grade ≥ 3, representing definite radiographic OA.

### Osteoporosis definitions

DXA-defined osteoporosis was defined as a T-score ≤ − 2.5 at any measured site (lumbar and/or FN), based on the lowest value across available sites for each patient. In addition, site-specific DXA-defined osteoporosis was categorized as lumbar osteoporosis (lumbar T-score ≤ − 2.5) and hip osteoporosis (FN T-score ≤ − 2.5 on either side). Hip osteoporosis was further classified according to laterality (operative/contralateral side).

Prevalent VFs were evaluated using Genant’s semi-quantitative (SQ) method by two independent assessors [22]. A VF was defined as an SQ grade ≥ 1 at any vertebra from T10 to L5. In cases of disagreement, a consensus was reached through discussion.

Overall, clinical osteoporosis was defined as meeting either the DXA-defined osteoporosis criteria or prevalent VFs.

### Statistical analysis

The distribution of patients according to DXA-defined osteoporosis and prevalent VFs was examined. Comparison between the contralateral normal and abnormal (OA or prior arthroplasty) groups was evaluated using a chi-square test. To explore clinical factors associated with VF without DXA-defined osteoporosis, multivariable logistic regression analysis was performed. Age (per 10-year increase), sex, body mass index (BMI; per 5-kg/m^2^ increase), and contralateral hip status were included as covariates based on clinical relevance. The odds ratios (ORs) with 95% confidence intervals (CIs) were calculated. As a sensitivity analysis, osteoporosis treatment at the time of surgical decision-making, including bisphosphonates, teriparatide, selective estrogen receptor modulators, and active vitamin D preparations, was included in the multivariable model.

Supplementary analyses described site-specific DXA osteoporosis patterns according to contralateral hip status (contralateral normal and contralateral OA). These analyses were restricted to patients for whom bilateral FN BMD data were available. The distribution of lumbar and FN osteoporosis was illustrated using Venn diagrams.

All analyses were performed using SPSS software (version 25.0; IBM Corp., Armonk, NY, USA).

## Results

A total of 391 consecutive patients who underwent primary THA were included. After excluding four patients who underwent THA following femoral neck fracture and 35 patients who underwent THA for non-OA etiologies (RA, *n* = 21; ION, *n* = 8; RDC, *n* = 5; SIF, *n* = 1), 352 patients remained in the final analysis. The baseline characteristics are presented in Table 1. The mean age was 68.9 years, and 299 patients (84.9%) were female. A total of 268 patients (76.1%) underwent THA for secondary OA due to DDH. Radiographically, 222 patients (63.1%) had normal contralateral hips, whereas 130 patients (36.9%) had abnormal contralateral hips (88 with OA change and 42 with prior arthroplasty).

**Table 1:** Patient characteristics

Characteristic	Total *N* = 352
**Age (years)**	68.9 ± 8.8
**Female, N (%)**	299 (84.9)
**Body Weight (kg)**	59.3 ± 12.3
**Height (cm)**	154.8 ± 7.4
**BMI (kg/m**^**2**^**)**	24.6 ± 4.3
**Etiology, N (%)**	
Secondary OA (DDH)	268 (76.1)
Primary OA	84 (23.9)
**Contralateral hip, N (%)**	
Normal	222 (63.1)
Abnormal	130 (36.9)
OA	88 (25.0)
Prior arthroplasty	42 (11.9)
**BMD**	
Lumbar spine (g/cm^2^)	1.038 ± 0.192
T-score	− 1.06 ± 1.32
Operated-side femoral neck (g/cm^2^)	0.862 ± 0.166
T-score	− 0.71 ± 1.46
Contralateral femoral neck (g/cm^2^)	0.783 ± 0.131
T-score	− 1.44 ± 1.23
**Osteoporosis treatment, N (%)**	65 (18.5)
**Activated Vitamin D, N (%)**	41 (11.6)
**Bisphosphonate**	23 (6.5)
**Others**	14 (4.0)

Data are presented as mean ± standard deviation

BMI, body mass index; OA, osteoarthritis; DDH, developmental dysplasia of the hip; BMD, bone mineral density

**Fig. 1:**
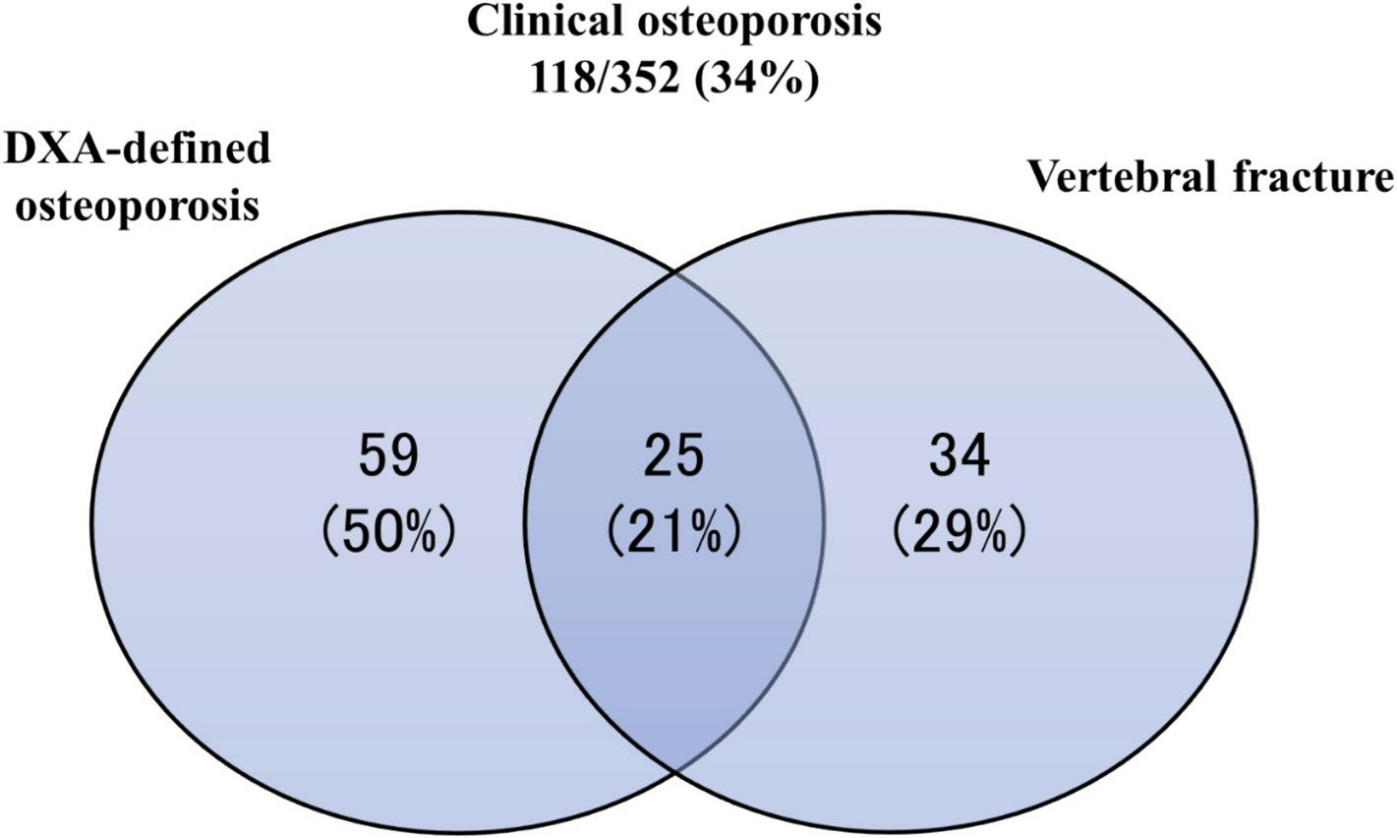
Overlap between dual-energy X-ray absorptiometry (DXA)-defined osteoporosis and vertebral fractures. Clinical osteoporosis was defined as DXA-defined osteoporosis or a prevalent vertebral fracture (VF). Numbers indicate the number of patients in each category, and percentages are calculated relative to the 118 patients with clinical osteoporosis. Of the 352 patients, 59 had DXA-defined osteoporosis alone, 25 had both DXA-defined osteoporosis and VFs, and 34 had VFs without DXA-defined osteoporosis

Table 2 summarizes the osteoporosis and prevalent VFs. DXA-defined osteoporosis was identified in 84 patients (23.9%), and prevalent VFs were observed in 59 patients (16.8%). Overall, 118 patients (33.5%) had clinical osteoporosis. Of these, 34 patients (9.7% of the total cohort; 28.8% of those with overall clinical osteoporosis) did not meet the DXA-defined osteoporosis criteria and would not have been identified using DXA alone (Fig. 1). When stratified according to the contralateral hip status (Table 2), there were no significant differences in any defined osteoporosis between the two groups. VF without DXA-defined osteoporosis was observed in 22 patients (9.9%) in the contralateral normal group and in 12 patients (9.2%) in the contralateral abnormal group.

**Table 2:** Osteoporosis status and comparisons between the contralateral normal and contralateral abnormal groups

Variables	Overall (*n* = 352)	Contralateral normal group (*n* = 222)	Contralateral abnormal group (*n* = 130)		*p*-value*
			**Contralateral OA (*n* = 88)**	**Contralateral arthroplasty (*n* = 42)**	
Clinical osteoporosis, *n* (%)	118 (33.5)	74 (33.3)	44 (33.8)		0.92
			29 (33.0)	15 (35.7)	
DXA-defined osteoporosis, *n* (%)	84 (23.9)	52 (23.4)	32 (24.6)		0.80
			22 (25.0)	10 (23.8)	
VF, *n* (%)	59 (16.8)	40 (18.0)	19 (14.6)		0.41
			11 (12.5)	8 (19.0)	
Both DXA-defined osteoporosis and VF, *n* (%)	25 (7.1)	18 (8.1)	7 (5.4)		0.34
			4 (4.5)	3 (7.1)	
VF without DXA-defined osteoporosis, *n* (%)	34 (9.7)	22 (9.9)	12 (9.2)		0.84
			7 (8.0)	5 (11.9)	
DXA-missed, % of clinical osteoporosis	28.8	29.7	27.3		
			24.1	33.3	

^*^p-values were calculated using the chi-square test for comparisons between the contralateral normal and abnormal groups

Clinical osteoporosis was defined as DXA-defined osteoporosis or prevalent vertebral fracture (VF). VF without DXA-defined osteoporosis was defined as the presence of VF in patients who did not meet DXA criteria for osteoporosis. DXA-missed cases were calculated as the proportion of patients with vertebral fractures without DXA-defined osteoporosis among those with clinical osteoporosis

DXA, dual-energy X-ray absorptiometry; OA, osteoarthritis; VF, vertebral fracture

**Fig. 2:**
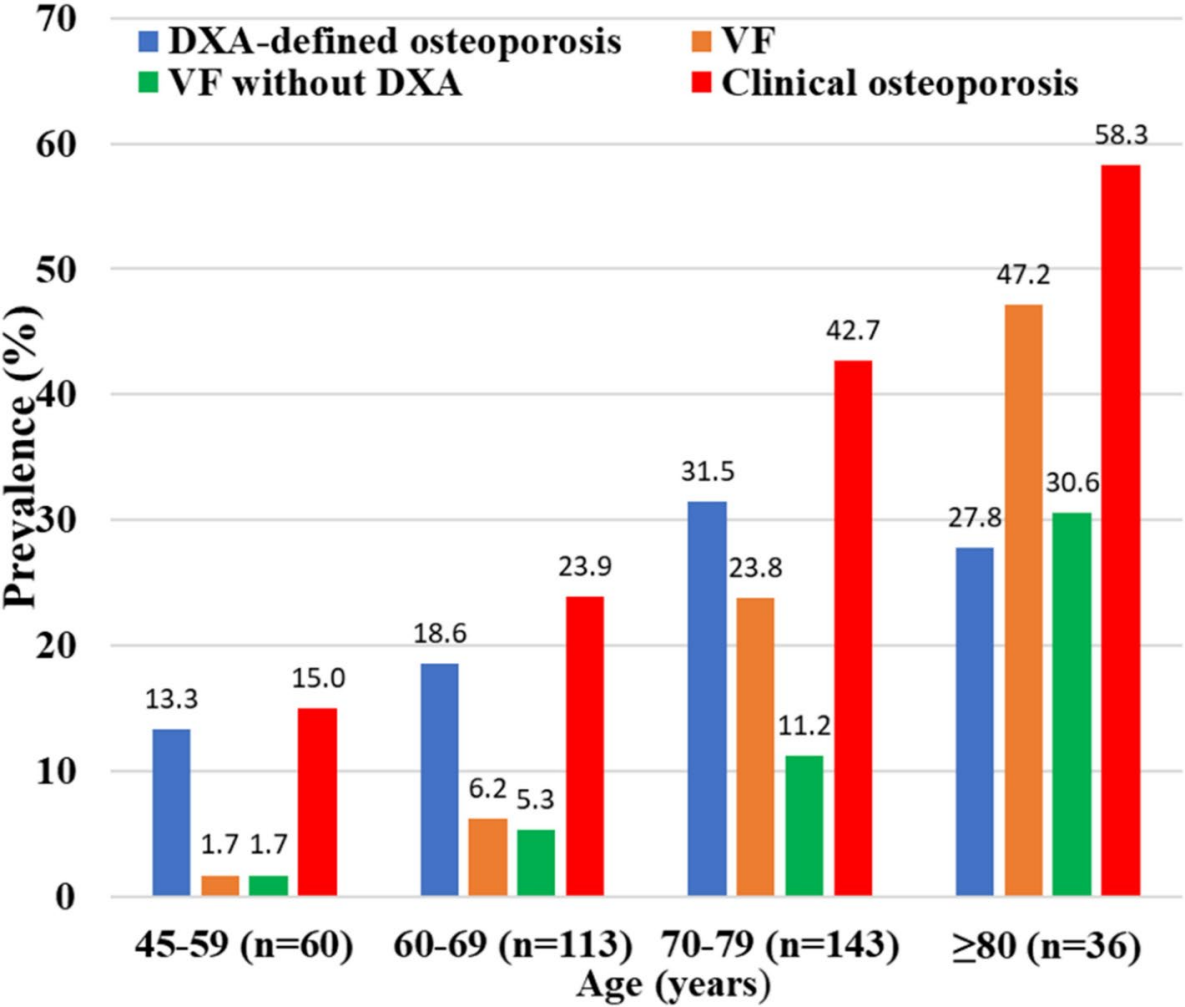
Age-stratified prevalence of dual-energy X-ray absorptiometry (DXA)-defined osteoporosis. Vertebral fracture (VF) without DXA-defined osteoporosis was defined as the presence of a VF in patients who did not meet the DXA criteria for osteoporosis. Clinical osteoporosis was defined as DXA-defined osteoporosis or a prevalent VF. The prevalence of VF, VF without DXA-defined osteoporosis, and clinical osteoporosis is shown across age groups

The results of the multivariable logistic regression analysis for VF without DXA-defined osteoporosis are shown in Table 3. Age was independently associated with VF without DXA-defined osteoporosis (adjusted OR 2.65 per 10-year increase; 95% CI 1.65–4.24; *p* < 0.001). The multivariable model demonstrated fair discrimination, with an area under the receiver operating characteristic curve of 0.720 (95% CI 0.627–0.813). This association remained unchanged after additional adjustment for current osteoporosis treatment (adjusted OR 2.40 per 10-year increase; 95% CI 1.47–3.91; *p* < 0.001). Age-stratified prevalence patterns are shown in Fig. 2. The prevalence of VF, clinical osteoporosis, and VF without DXA-defined osteoporosis increased with age, whereas DXA-defined osteoporosis changed less across age groups. The prevalence of VF without DXA-defined osteoporosis was 30.6%, and that of clinical osteoporosis was 58.3% in patients aged ≥ 80 years.

**Table 3:** Multivariable logistic regression for VF without DXA-defined osteoporosis

Variables	VF without DXA, *n* = 34	Others, *n* = 318	Adjusted OR (95% CI)	*p*-value
Age (years)	74.7 ± 8.4	68.2 ± 8.7	2.65 (1.65–4.24) †	< 0.001
Female, *n* (%)	27 (79.4)	272 (85.5)	0.57 (0.22–1.43)	0.23
BMI (kg/m^2^)	24.9 ± 4.3	24.6 ± 4.3	1.26 (0.80–1.97) ‡	0.32
Contralateral abnormal, *n* (%)	12 (35.3)	118 (37.1)	0.98 (0.46–2.11)	0.96

^†^per 10-year increase; ‡per 5-kg/m^2^ increase

Data are presented as mean ± standard deviation

VF, vertebral fracture; DXA, dual-energy X-ray absorptiometry; OR, odds ratio; CI, confidence interval; BMI, body mass index

**Fig. 3:**
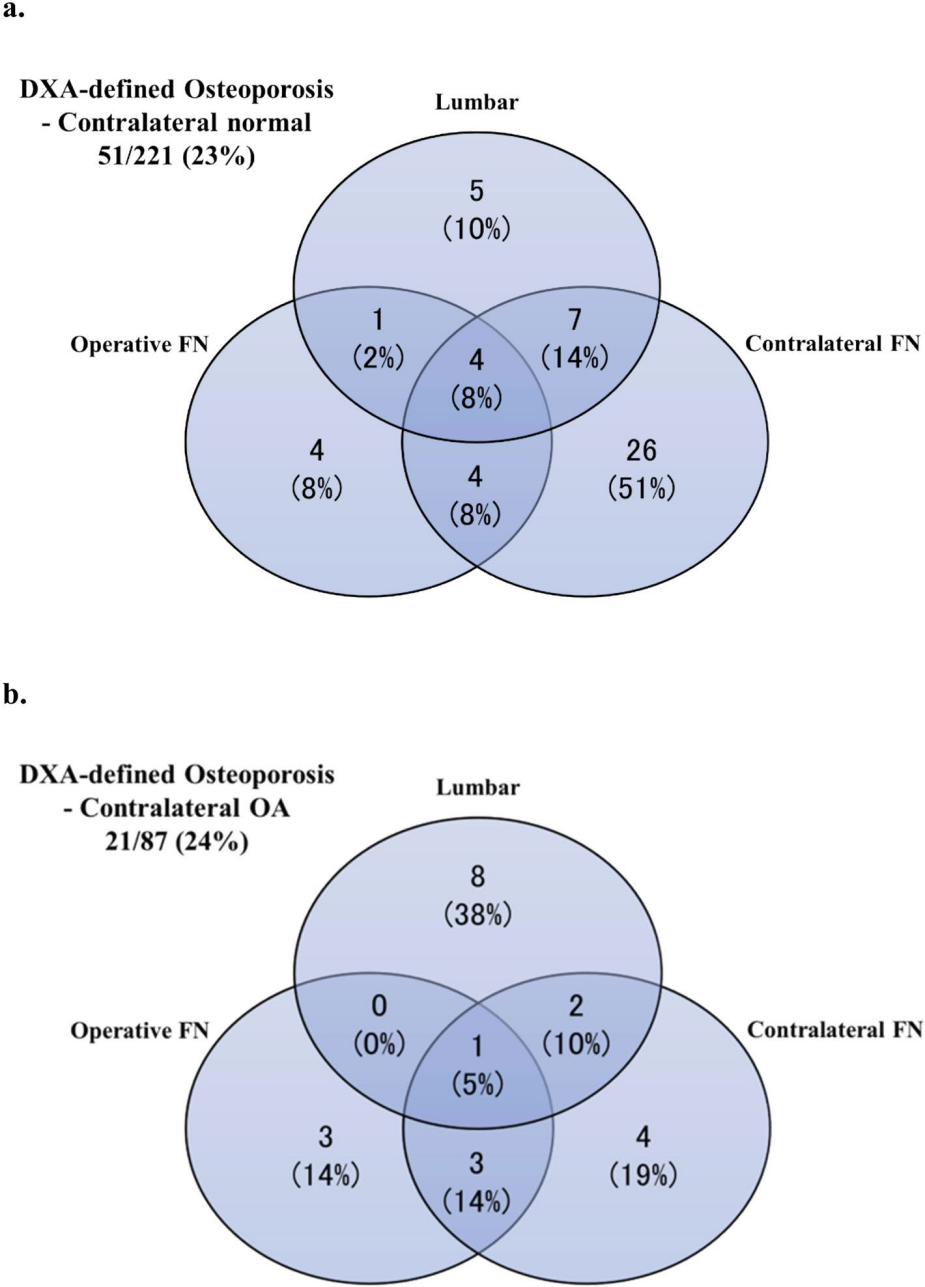
Overlap of site-specific osteoporosis defined by dual-energy X-ray absorptiometry (DXA). Venn diagrams show the overlap of site-specific DXA-defined osteoporosis among patients for whom bilateral femoral neck (FN) bone mineral density (BMD) measurements were available: (a) the contralateral normal group and (b) the contralateral osteoarthritis (OA) group. Numbers indicate the number of patients in each category, and percentages are calculated relative to the patients with DXA-defined osteoporosis in each group

The distribution of site-specific DXA-defined osteoporosis among patients with lumbar and bilateral FN BMD is shown in Fig. 3. In the contralateral normal group (*n* = 221), among patients classified as having DXA-defined osteoporosis (*n* = 51), 26 (51.0%) showed reduced BMD exclusively at the contralateral FN, whereas only 4 (7.8%) showed reduced BMD exclusively at the operative FN. In contrast, in the contralateral OA group (*n* = 87), among patients classified as having DXA-defined osteoporosis (*n* = 21), 4 (19.0%) showed reduced BMD exclusively at the contralateral FN, whereas 3 (14.3%) showed reduced BMD exclusively at the operative FN.

## Discussion

In our cohort, the incorporation of VF evaluation substantially altered the identification of bone fragility compared with DXA-based assessment alone. The prevalence of DXA-defined osteoporosis was 23.9%, which is comparable to the prevalence previously reported in THA cohorts [20, 21]. However, when radiographic VF assessment was incorporated, the prevalence of clinical osteoporosis increased to 33.5%. Notably, reliance on DXA alone would have failed to identify nearly one-third of patients with evidence of bone fragility. This underestimation was observed irrespective of contralateral hip status. To the best of our knowledge, this is the first study to evaluate both DXA-defined osteoporosis and prevalent VFs in a THA population. These findings indicate that DXA-based classification alone systematically underestimates bone fragility in patients undergoing THA. Therefore, comprehensive evaluation of bone fragility, including VF assessment, may contribute to perioperative osteoporosis management and support preoperative risk assessment.

Although areal BMD measured by DXA represents only one component of bone strength, it is a well-established predictor of fracture risk [23]. For instance, the deterioration of bone microarchitecture, such as trabecular number, trabecular separation, and connectivity density, which cannot be measured by DXA, is associated with VFs independent of BMD [24, 25]. VFs are also independent risk factors for subsequent fragility fractures. Prior VFs have been reported to increase the risk of subsequent VFs by approximately fourfold in men and threefold in women, even after adjusting for age and baseline BMD [26]. Similarly, in a large 15-year longitudinal cohort study, Cauley et al. demonstrated that both low BMD and the presence of prevalent VFs were independently associated with incident VFs [27]. These findings indicate that assessing VF status provides clinically meaningful information. In several countries, including Japan, the presence of VF is explicitly incorporated into the diagnostic or therapeutic criteria for osteoporosis, reflecting the recognition of fracture-based diagnosis as an essential component of fragility assessment [10, 28, 29]. Importantly, many VFs are clinically silent and remain unrecognized without radiographic evaluation [15–17]. Therefore, the assessment of bone fragility should not rely solely on BMD but should also incorporate radiographic evaluation of VFs.

In the context of THA, several studies demonstrated an association between preoperative fragility fractures and adverse outcomes after THA. Using a large administrative claims database, Ross et al. reported that fragility fractures within three years prior to THA were associated with an approximately 1.8-fold higher risk of PPF and a threefold higher risk of subsequent fragility fractures within 2 years after THA [30]. Furthermore, similar findings were reported in a study with up to 8 years of follow-up [31]. Thus, preoperative evaluation of prevalent fractures is clinically important. A position statement by a group of seven national societies stated that bone health assessments should be performed for patients scheduled for elective arthroplasty [32]. Nevertheless, systematic evaluation of VFs before THA remains limited. In our cohort (mean age 68.9 years; 84.9% female), VFs were identified in approximately 17% of patients. This prevalence is comparable to age-matched population-based data reported in women [12]. Chen et al. reported that 11.3% of patients undergoing THA had VFs, and those with VFs had poorer clinical outcomes at 1 year postoperatively than those without VFs [33]. However, in their analysis, VFs were not integrated with the BMD-based assessment. Although the prevalence of VFs in their cohort was lower than that in our cohort, possibly reflecting their relatively younger population, both studies suggest that VFs are not rare in the THA population.

In our study, 9.7% of patients undergoing THA did not meet the DXA-defined osteoporosis criteria but had VF, and advanced age was independently associated with VF without DXA-defined osteoporosis. In the multivariable analysis, each 10-year increase in age was associated with nearly a threefold higher likelihood of having VF without DXA-defined osteoporosis. Age-stratified analysis supported this finding; VF without DXA-defined osteoporosis was more frequently observed in patients aged ≥ 70 years and was particularly common in those aged ≥ 80 years. Among patients aged ≥ 80 years, clinical osteoporosis was present in approximately 60% of patients, whereas VF without DXA-defined osteoporosis was observed in approximately 30%. One possible explanation is the effect of osteoporosis treatment, which increases BMD in patients with VF. However, a similar association was observed after adjusting for osteoporosis treatment. In a previous observational study, age itself was shown to be a strong predictor of VF even in the absence of osteoporosis, and a discrepancy between VF incidence and BMD was highlighted [34]. As noted above, various factors related to bone fragility not assessed by DXA may worsen with age. In addition, in older patients, structural alterations such as osteophyte formation, vascular calcification, and even the presence of VFs may mask underlying bone fragility when assessed using lumbar BMD [35, 36]. From a clinical standpoint, preoperative VF assessment should ideally be performed in all patients. In resource-limited settings, prioritizing such evaluations in older patients may be a pragmatic and clinically appropriate approach.

The distribution of sites diagnosed with osteoporosis using DXA differed depending on the presence of contralateral OA. In the contralateral normal group, 51% of DXA-defined osteoporosis cases were identified only at the contralateral FN (healthy side) rather than at the lumbar or operative FN. This finding suggests that approximately half of DXA-defined osteoporosis cases may remain undetected if only operative side BMD is measured. This pattern is consistent with those reported in previous studies [37, 38]. Uemura et al. assessed bilateral hip BMD from CT images in patients with unilateral hip OA and reported that osteoporosis diagnoses based on the OA side had a lower sensitivity than those based on the healthy side (61% vs. 88%) [37]. In contrast, in the contralateral OA group, the overall prevalence of DXA-defined osteoporosis was nearly equivalent to that in the contralateral normal group. Notably, there were relatively more cases in which only the lumbar region was DXA-positive (38%), whereas no significant side-to-side differences were observed between the hips. The contralateral OA group (KL ≥ 3) may not represent a homogeneous skeletal phenotype. This subgroup likely includes patients with true low bone mass and bone fragility as well as those with an osteophyte-/sclerosis-dominant OA phenotype, in whom areal hip BMD may appear relatively preserved or even elevated. Such heterogeneity may obscure clear side-specific trends in hip BMD. From a practical perspective, these findings suggest that in unilateral hip OA, assessment of the contralateral FN BMD is particularly informative, whereas in patients with bilateral hip OA, bilateral FN BMD measurements, in addition to lumbar spine BMD, may be preferable when feasible.

This study has some limitations. First, the VF assessment was limited to the T10–L5 levels, and information on fragility fractures at other skeletal sites was not available. Ideally, a more comprehensive fracture assessment, including the upper thoracic spine and non-vertebral fragility fractures, should be incorporated when evaluating bone fragility. Therefore, the overall prevalence of bone fragility might have been underestimated. Second, the hip BMD assessment was based only on FN measurements, without total hip measurements. In patients with hip OA, accurate FN BMD assessment may be affected by positioning difficulties or restricted range of motion. However, because FN measurement remains the standard in many DXA systems, our approach may also reflect real-world clinical practice. Third, this was a single-center study involving a single surgical cohort. However, it would be reliable due to evaluation using single-source DXA. Finally, postoperative outcomes were not evaluated in this study. Therefore, whether prevalent VFs are associated with complications related to bone fragility such as PPF remains unclear. Future prospective longitudinal studies are needed to determine the prognostic significance of VF in patients undergoing THA.

## Conclusion

The prevalence of clinically relevant bone fragility was at least 33.5% when VFs were considered, corresponding to clinical osteoporosis. Nearly one-third of clinical osteoporosis cases in patients undergoing THA were not identified by DXA criteria alone. Thus, reliance solely on BMD assessment may underestimate bone fragility. Given that older patients are at greater risk of VFs even without DXA-defined osteoporosis, preoperative VF assessment should be considered, particularly in patients aged ≥ 70 years. Incorporating preoperative spine radiographs may improve the assessment of bone fragility in patients undergoing THA.

## Data Availability

The datasets used and/or analyzed during the current study are available from the corresponding author on reasonable request.

## Abbreviations

BMD: Bone mineral density
BMI: Body mass index
CI: Confidence interval
DDH: Developmental dysplasia of the hip
DXA: Dual-energy X-ray absorptiometry
FN: Femoral neck
JSBMR: Japanese Society for Bone and Mineral Research
KL: Kellgren–Lawrence
OA: Osteoarthritis
OR: Odds ratio
PPF: Periprosthetic femoral fracture
SQ: Semi-quantitative
THA: Total hip arthroplasty
VF: Vertebral fracture
